# *IL18*-containing 5-gene signature distinguishes histologically identical dermatomyositis and lupus erythematosus skin lesions

**DOI:** 10.1172/jci.insight.139558

**Published:** 2020-08-20

**Authors:** Lam C. Tsoi, Mehrnaz Gharaee-Kermani, Celine C. Berthier, Tori Nault, Grace A. Hile, Shannon N. Estadt, Matthew T. Patrick, Rachael Wasikowski, Allison C. Billi, Lori Lowe, Tamra J. Reed, Johann E. Gudjonsson, J. Michelle Kahlenberg

**Affiliations:** 1 Department of Dermatology and; 2 Department of Computational Medicine & Bioinformatics, University of Michigan Medical School, Ann Arbor, Michigan, USA.; 3 Department of Biostatistics, School of Public Health, University of Michigan, Ann Arbor, Michigan, USA.; 4 Division of Rheumatology, Department of Internal Medicine,; 5 Division of Nephrology, Department of Internal Medicine,; 6 Division of General Medicine, Department of Internal Medicine,; 7 Program in Immunology, and; 8 Department of Pathology, University of Michigan Medical School, Ann Arbor, Michigan, USA.

**Keywords:** Autoimmunity, Dermatology, Autoimmune diseases, Cytokines, Skin

## Abstract

Skin lesions in dermatomyositis (DM) are common, are frequently refractory, and have prognostic significance. Histologically, DM lesions appear similar to cutaneous lupus erythematosus (CLE) lesions and frequently cannot be differentiated. We thus compared the transcriptional profile of DM biopsies with CLE lesions to identify unique features. Type I IFN signaling, including IFN-κ upregulation, was a common pathway in both DM and CLE; however, CLE also exhibited other inflammatory pathways. Notably, DM lesions could be distinguished from CLE by a 5-gene biomarker panel that included *IL18* upregulation. Using single-cell RNA-sequencing, we further identified keratinocytes as the main source of increased IL-18 in DM skin. This study identifies a potentially novel molecular signature, with significant clinical implications for differentiating DM from CLE lesions, and highlights the potential role for IL-18 in the pathophysiology of DM skin disease.

## Abstract 2

IL-18 distinguishes dermatomyositis skin inflammation from cutaneous lupus erythematosus lesions.

## Introduction

Dermatomyositis (DM) is an autoimmune disease characterized by a skin rash and accompanied by prominent muscle weakness. Cutaneous manifestations can follow a polyphasic, relapsing course (1) and possibly predict systemic manifestations (2). Full clinical remission of skin lesions can be difficult to achieve, reflecting a poor understanding of the pathogenesis of skin lesions in this disease. Further, histopathologic factors common to cutaneous DM lesions, including vacuolar interface dermatitis, perivascular inflammation, increased dermal mucin, and dyskeratotic keratinocytes (3), are also seen in cutaneous lupus erythematosus (CLE) lesions, making DM-associated skin eruptions difficult to distinguish from CLE, especially in the absence of obvious muscle disease or lesional pathognomonic patterns. There has been very limited research published on the molecular differences between DM and CLE, and no biomarker has yet been identified to differentiate the 2 conditions.

Similar to systemic lupus erythematosus (SLE) and CLE, research on DM pathogenesis has identified a strong type I IFN blood signature that correlates with the severity of skin activity (4, 5). Additionally, type I IFNs are upregulated in lesional skin (6) and are thought to play a pathogenic role in destruction of muscle tissue (7). Further, previous research examining the roles of B cells, T cells, and DCs has focused primarily on muscle pathology (reviewed in ref. 8). However, the inflammatory cell contributions in DM skin have not yet been thoroughly examined.

In this study, we investigate the transcriptional changes in a large cohort of DM (43 lesional skin samples from 36 DM patients) and compare them with CLE biopsies to identify distinguishing features between DM and CLE lesions. Although we confirm previous observations that type I IFN signatures predominate in DM, we also identify IFN-κ upregulation in the epidermis as a common IFN signal between DM and CLE. Notably, we report a DM-specific 5-gene signature that, in combination, distinguishes DM from CLE, further validated in independent samples. These results suggest that IL-18 is a novel player in DM skin disease and illustrate its potential clinical impact to serve as a biomarker, differentiating DM and CLE.

## Results

**Figure 1:**
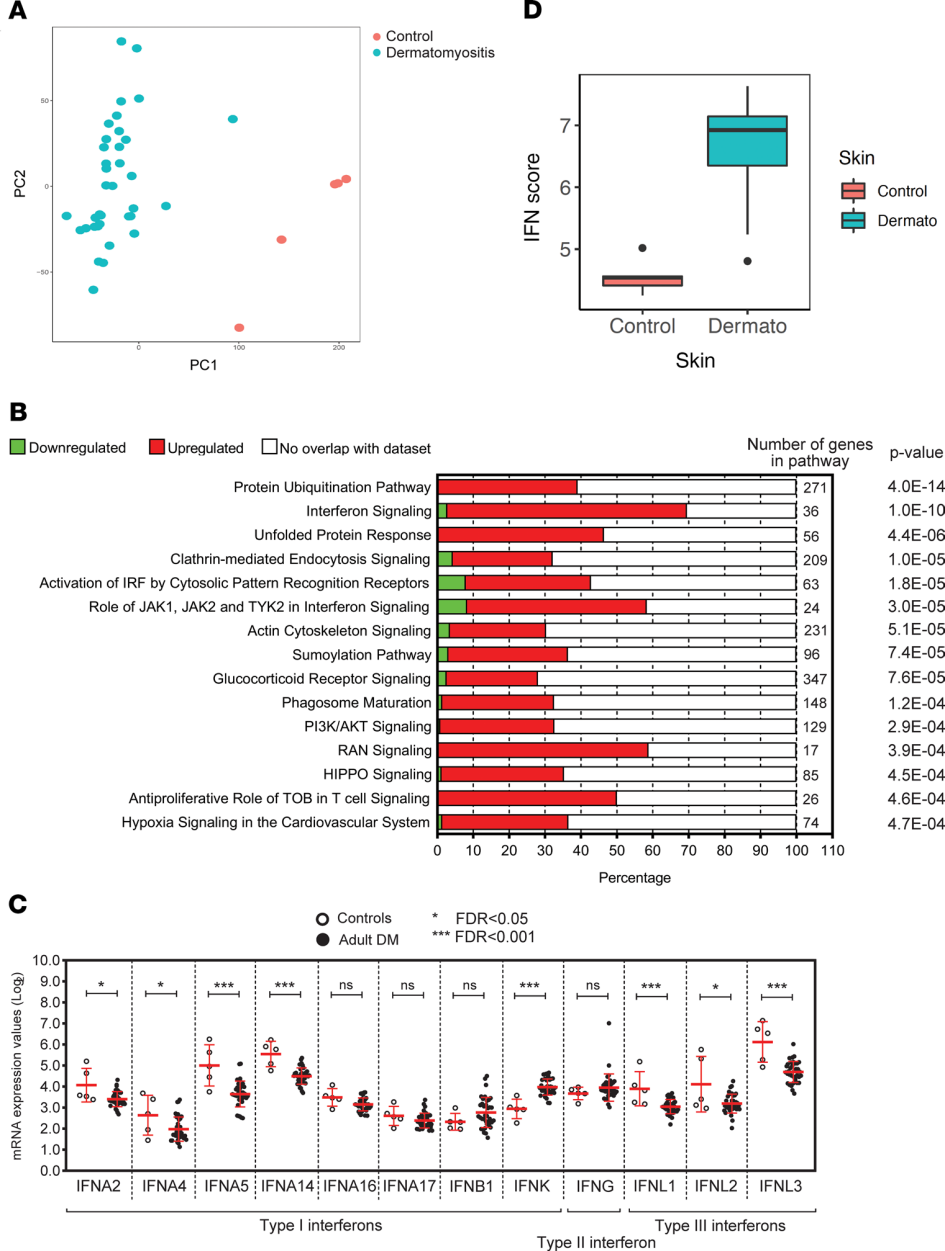
DM skin lesions demonstrate a strong IFN signature. (**A**) Principal components analysis of differentially expressed genes (DEGs) between lesional DM biopsies and healthy control skin. (**B**) IPA identifying key pathways in the DM DEG list. (**C**) Graphical representation of log_2_ mRNA expression values of IFN genes from DM lesional skin microarrays. Bars depict SD. (**D**) IFN score comparison between DM and healthy control biopsies. The box plots depict the minimum and maximum values (whiskers), the upper and lower quartiles, and the median. The length of the box represents the interquartile range.

A total of 43 cutaneous lesional biopsies from 36 validated DM patients were collected from the University of Michigan (U-M) Archives of Pathology & Laboratory Medicine (Table 1). Comparison of DM lesional samples with healthy control skin (*n* = 5) identified 6382 differentially expressed genes (DEGs): 3398 of which were upregulated and 2984 of which were downregulated, respectively, in DM. Principal components analysis identified clear separation of DM cases from healthy controls (Figure 1A). Evaluation of DEGs using Ingenuity Pathway Analysis (IPA; QIAGEN) identified protein ubiquitination and IFN signaling as the top 2 differentially regulated pathways (Figure 1B).

**Figure 2:**
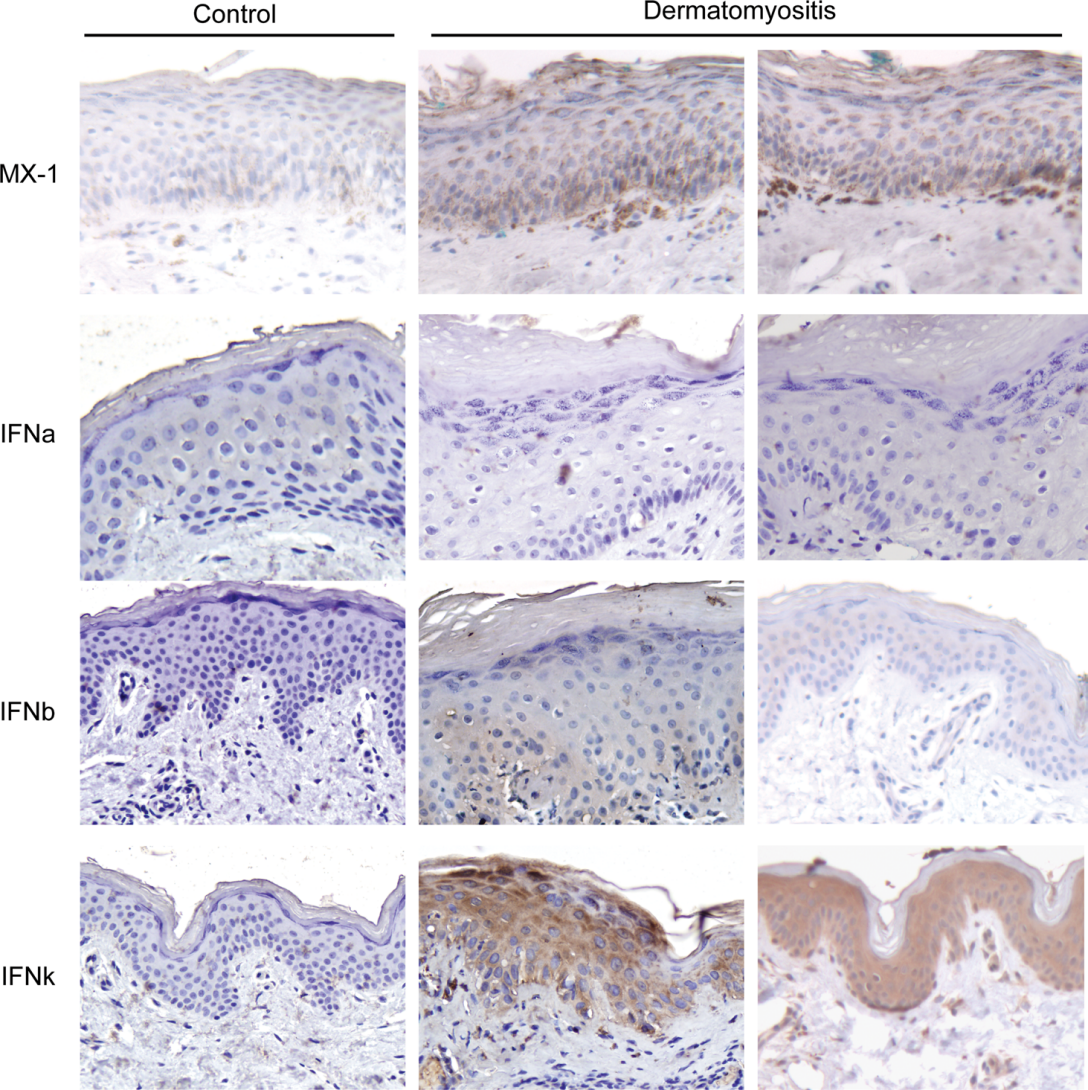
IFN-β and IFN-κ protein expression are increased in DM skin. IHC of healthy control or 2 DM lesional biopsies (representative out of 8 patients) for the IFN-regulated protein MX1 and IFN-α, IFN-β, and IFN-κ. Original magnification, ×20.

Based on previous work, IFN-regulated gene expression was expected to be elevated in DM lesions (6), and recently, IFN-κ has been identified as an important type I IFN in CLE epidermis (9). Given that DM skin also demonstrated elevated IFN signatures, we evaluated subtypes of IFN expression in DM skin. IFN-κ transcription was upregulated (*P* *=* 1.3 × 10^–6^) in DM lesional skin (Figure 1C). IFN-β expression was also increased in DM lesions but was not statistically significant. To confirm IFN-regulated gene upregulation in the DM lesions, we next computed IFN scores using the IFN-inducible genes previously reported (6) and demonstrated that DM samples have significantly higher IFN “burden” (*P* = 5.3 × 10^–6^) than control samples (Figure 1D). IHC of DM lesions further confirmed increased MX1 staining (as a marker of IFN exposure), increased IFN-κ staining, and some increase in IFN-β staining (Figure 2). Additionally, IFN-α expression was not detected by IHC in DM lesional skin (Figure 2). Therefore, these data support IFN-κ and IFN-β upregulation as contributors to the IFN signature in DM skin lesions.

**Figure 3:**
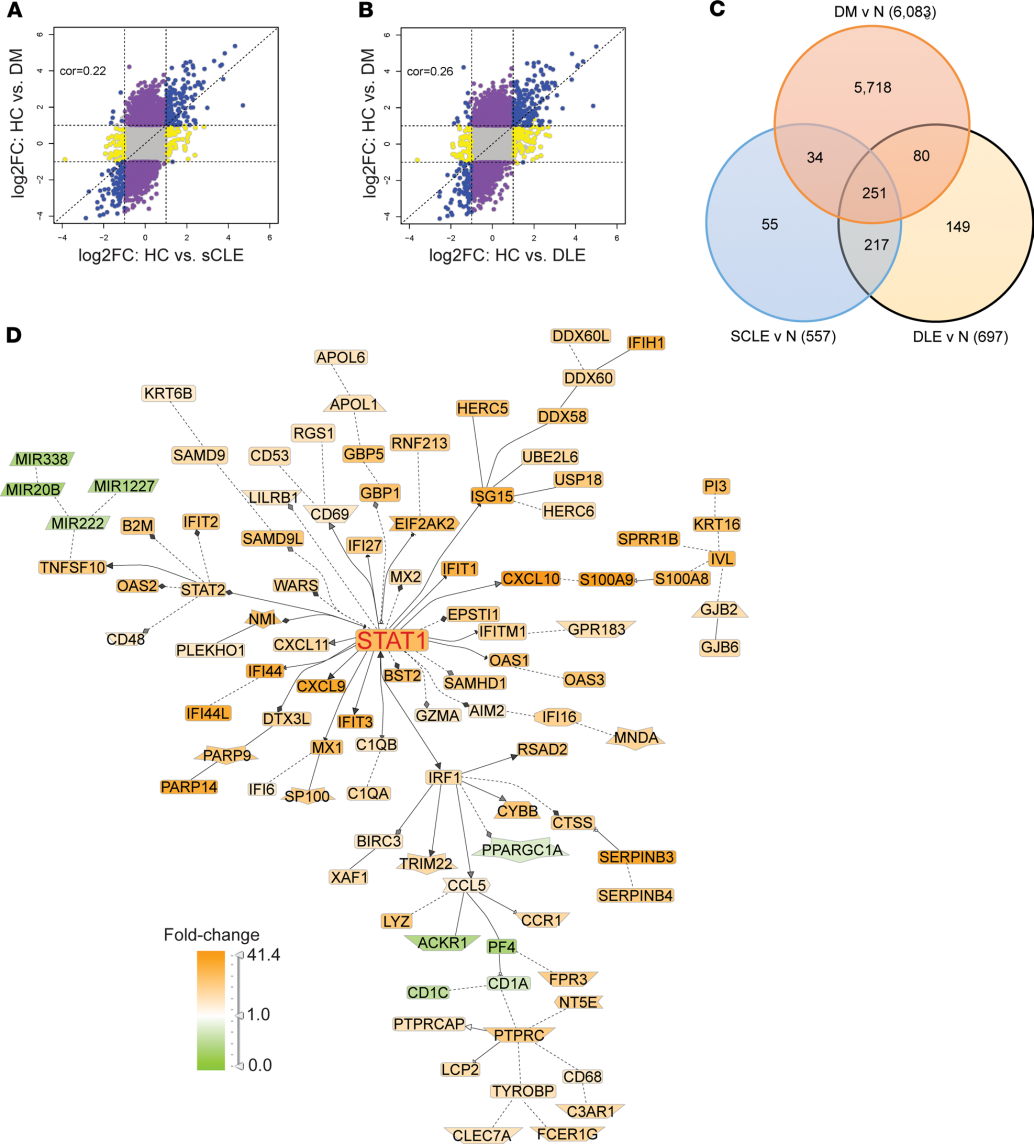
DM shares IFN genes with CLE lesions. (**A** and **B**) Comparison of DEGs in DM lesional skin (*y* axis) versus DEGs in SCLE (**A**) and DLE (**B**). Shared DEGs in the same direction are denoted in blue. (**C**) Venn diagram showing shared and unique DEGs between DM, SCLE, and DLE. (**D**) Genomatix Pathway analysis of shared 251 DEGs between DM and CLE subtypes.

**Figure 4:**
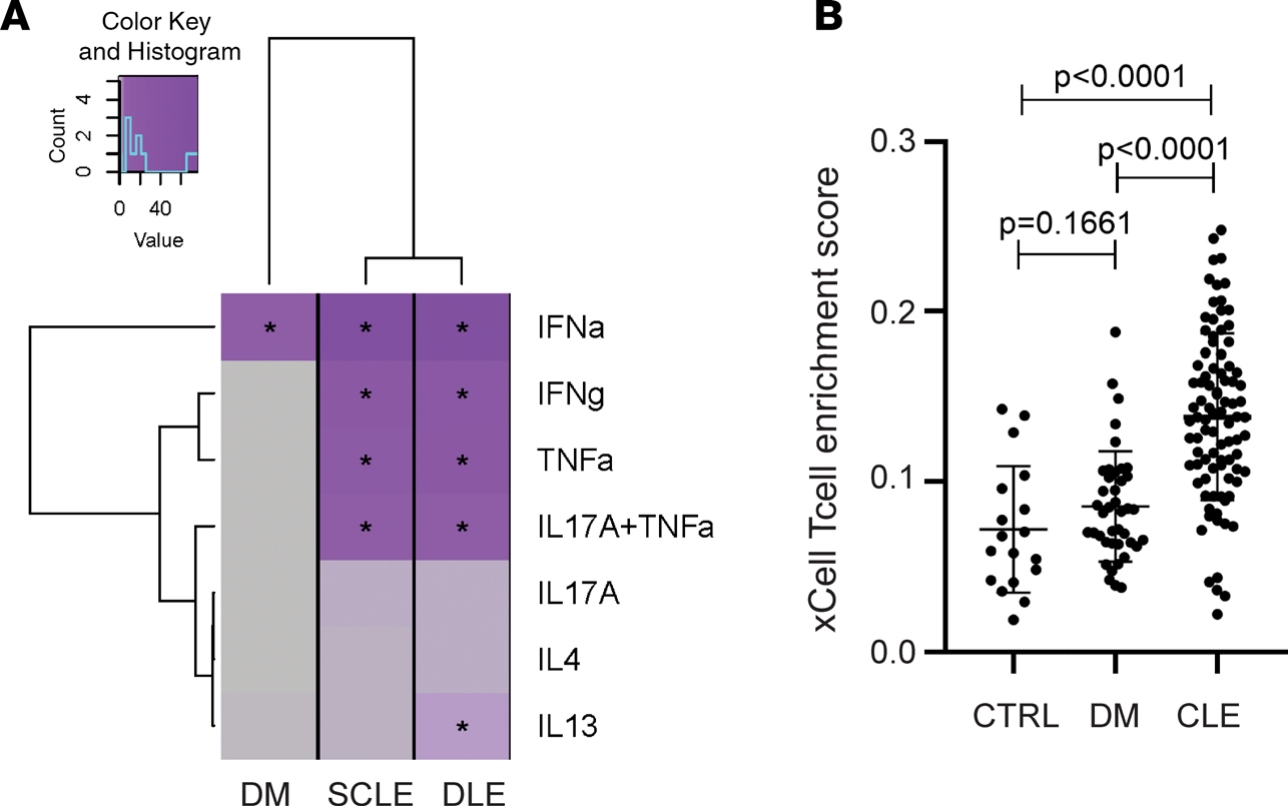
DM exhibits fewer T cell–related genes compared with CLE. (**A**) Heatmap of overlap of DM, SCLE, and DLE lesional DEGs with cytokine signatures generated via stimulation of keratinocytes with indicated cytokines followed by RNA-Seq. (**B**) xCell analysis shows no increase in total T cell score (driven by CD4^+^ central memory, effector memory, memory, and naive cells and CD8^+^ central memory, effector memory, and naive cells) in DM lesions versus healthy control (HC) whereas CLE has a high T cell score. Bars depict SD.

Given the similarity in IFN gene upregulation between DM and CLE, we next compared the overlap of DM DEGs with those seen in CLE subtypes (chronic cutaneous or discoid lupus erythematosus [DLE] and subacute cutaneous lupus erythematosus [SCLE]). There was significant enrichment of SCLE (*P* *=* 1.2 × 10^–64^) and DLE (*P* *=* 2 × 10^–61^) DEGs among the DEGs in the DM biopsies, and dysregulation between DM and SCLE/DLE was also highly correlated (Figure 3, A and B). Evaluation of DEGs shared between DM, DLE, and SCLE identified 251 genes, including 244 with the same directionality in all 3 diseases (Figure 3C and Supplemental Table 1; supplemental material available online with this article; https://doi.org/10.1172/jci.insight.139558DS1). Literature-based network analysis identified these 251 shared genes as primarily centered on IFN signaling, as demonstrated by the central *STAT1* node (Figure 3D). In addition, the 3 significantly regulated transcription factors in this shared gene subset were *STAT1*, *IRF1*, and *STAT2* as assessed by transcription factor analysis (Supplemental Table 2), consistent with a shared IFN signal between DM and both CLE subtypes. To identify if these overlapping signatures represented other pathways beyond type I IFNs, we then compared DEGs of DM and CLE lesions with epidermal cytokine signatures generated via RNA-sequencing (RNA-Seq) of cytokine-stimulated keratinocytes (10). Surprisingly, whereas SCLE and DLE displayed DEGs consistent with TNF, IFN-γ, and type I IFN stimulation, DM lesions exhibited overlap with only type I IFN–mediated changes (Figure 4A). These data suggest that DM skin lesions may represent a more homogeneous type I IFN disease characterized by less inflammation and less T cell activation. In fact, microarray data analysis by xCell, which assigns cell type enrichment scores using gene expression data (11), demonstrated a lower T cell score for DM when compared with CLE (Figure 4B and Supplemental Figure 1).

**Figure 5:**
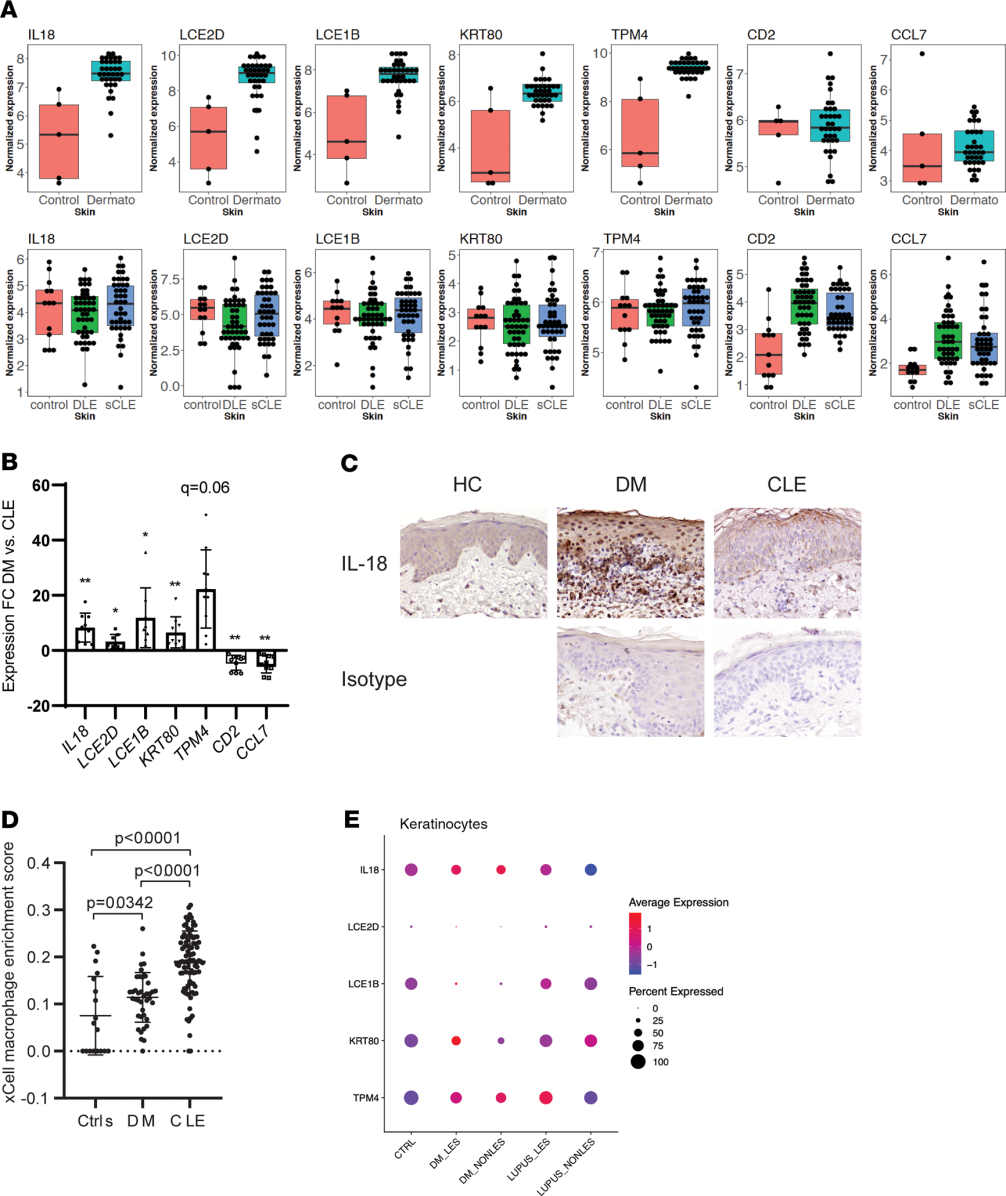
DM lesions can be distinguished by a 5-gene score and exhibit increased IL-18 in dermal inflammation. (**A**) Box plots of DEGs in DM but not CLE (*IL18* and *LCE2D*) or DEGs in CLE but not DM (*CCL7* and *CD2*). (**B**) RNA was isolated from 9 DM and 9 CLE lesional samples from the independent validation cohort and subjected to real-time PCR with the indicated primers. Data are presented as the fold change calculated as 2^-ΔΔCT^ of DM versus CLE. Statistical significance was calculated via multiple 2-tailed *t* tests using false discovery rate to account for multiple comparisons of delta CT values normalized to *GAPDH* expression. The *q* values are denoted on the graph as follows: *q < 0.05; **q < 0.01. (**C**) IHC for IL-18 or isotype control in healthy control (representative of 3 controls), DM (representative of 3 patients), and CLE skin (representative of 4 patients). (**D**) xCell enrichment score for macrophage-derived transcripts in HC, DM, and CLE lesions. (**E**) scRNA-Seq analysis of DM lesional and nonlesional skin compared with healthy control and lupus skin. Graph represents gene expression in keratinocytes for percentage of cells expressing the indicated gene (by circle size) and degree of expression (by color).

Given these differences between DM and CLE, and the large number of DEGs that were noted to be unique to DM (5718; Figure 3C), we then evaluated whether a combination of DEGs in DM skin could distinguish DM from CLE, which to date has not yet been accomplished by expert pathologists with H&E evaluation of lesional tissue. We performed classification analysis using the top upregulated genes in DM samples, restricting DEGs identified in DM samples not differentially regulated in SCLE (with the defined criteria 0.67 < fold change < 1.5 and *P* > 0.5) (examples in the first 5 genes, Figure 5A). Using a gene panel consisting of transcripts involved in immune-related responses (*IL18*, *LCE2D*, *LCE1B*, *KRT80*, and *TPM4*), we were able to achieve an area under the curve (AUROC) of 0.98 to discriminate DM from SCLE using a 5-fold cross-validation. Similar results were achieved via comparison with DLE lesions (AUROC = 0.98). We thus termed this 5-gene signature as the DM biomarker panel. Figure 5A shows a comparison of expression versus control for the 5-gene signature. Genes skewed toward CLE but not DM, such as *CCL7* and *CD2*, were also identified, but these did not contribute to improvement of ROC curve (Figure 5A). We next validated this 5-gene signature on a new independent cohort of 9 DM and 9 CLE lesional samples via real-time PCR. Figure 5B shows that *IL18*, *LCE2D*, *LCE1B*, *KRT80*, and *TPM4* expression were significantly elevated in DM when compared with CLE lesions in the validation cohort. To follow up on the role of IL-18 in DM skin, we then looked at protein expression by IHC. Figure 5C shows that by IHC, DM lesions exhibited elevated expression of IL-18, with most located in the dermal inflammatory infiltrate and keratinocytes. xCell analysis suggested a highly significant increase in macrophage signatures in DM skin, purporting monocytes and/or macrophages as a potential inflammatory source of this cytokine (Figure 5D). We next used scRNA-Seq to examine *IL18* expression in 2 control, 2 paired lesional and nonlesional biopsies from DM patients, and 2 paired lesional and nonlesional biopsies from 2 lupus patients with SCLE. Figure 5E shows that keratinocytes expressed IL-18, and both lesional and nonlesional DM keratinocytes exhibited increased *IL18* expression over control and lupus biopsies. Similarly, by profiling the other 4 genes in the panel, all except TPM4 demonstrated higher average expression in DM keratinocytes. Additionally, no differences in IL-18 expression were noted in myeloid cells between DM and SLE (Supplemental Figure 2). In summary, these data suggest that in DM lesions, a 5-gene signature distinguishes CLE from DM skin lesions and that keratinocytes may be driving detectable differences between CLE and DM, especially for *IL18*.

## Discussion

DM skin lesions can be challenging to treat (12) and may be difficult to distinguish from cutaneous lupus lesions by biopsy. This study used a large cohort of DM skin lesions to identify transcriptional changes in the skin to generate a better understanding of disease pathology and to provide clues for improved treatment of refractory skin lesions. In addition, comparison with 90 CLE biopsies identified a 5-gene signature that can distinguish CLE from DM lesions.

IFNs have been identified as important in both muscle and skin disease in patients with DM. An increased type I IFN signature was also identified using oligonucleotide arrays on 16 lesional DM skin biopsies (6). In that study, IFN-β strongly correlated with the IFN signature in the skin. However, an increase in IFN-κ was unable to be identified with their custom array. Here, we identified a significant increase in IFN-κ in DM lesions by microarray, real-time PCR, and IHC. We also detected a trend for increased IFN-β in DM lesions. This suggests that IFN-κ and IFN-β may both have important roles in DM lesions.

The impact of IFN on DM skin is not well understood. Melanoma differentiation-associated protein 5 (MDA5) antibody–positive (MDA5^+^) patients had the highest serum IFN levels when compared with DM patients with anti–aminoacyl-tRNA synthetase antibodies or no autoantibody positivity (13). Interestingly, anti-MDA5 antibodies were also associated with the most refractory skin findings in 1 cohort of 74 patients (12). Our cohort, however, did not have sufficient MDA5^+^ patient numbers to analyze correlation with IFN in the skin. In lupus keratinocytes, elevated IFNs promote IL-6 production following TLR or UVB stimulation of keratinocytes (14) and also promote photosensitive responses (9) and disruption of the epidermal barrier (15). DM skin is known to be photosensitive, and total UV exposure is associated with a higher risk of developing DM (16). Whether IFNs generate photosensitive responses in DM skin should be the subject of further investigation.

Serum cytokines have also been evaluated as biomarkers in DM patients. Serum IFN-β and CXCL10 levels correlate with the Cutaneous Dermatomyositis Disease Area and Severity Index activity score. Serum IL-18, IL-6, IL-10, and IFN-β were increased in DM patients with interstitial lung disease as well (17). However, until our study, no data set to our knowledge has identified cutaneous biomarkers specific to DM that are able to distinguish it from its histologic twin, CLE. Intriguingly, some of these biomarkers (*LCE2D*, *LCE1B*, and *KRT80*) likely reflect the hyperproliferation and/or hypersquamatization of the epidermis that can been seen in DM lesions (18). Both *LCE2D* and *LCE1B* are part of the late cornified envelope and would be present in higher amounts in hyperkeratotic lesions. *KRT80* encodes a type II keratin also involved in terminal differentiation of the epithelium. *TMP4* encodes tropomyosin 4, a protein that interacts with the cytoskeleton in nonmuscle cells and regulates calcium flux. Notably, tropomyosin family members are capable of inducing autoimmune responses (19, 20) and have been reported as targets of autoantibodies in dermatomyositis patients (21).

IL-18 has previously been identified to be elevated in DM muscle biopsies, and expression decreases with immunosuppressive treatment for the muscle disease (22). In the skin, activation of IL-18 has been identified as important in other autoimmune and inflammatory skin diseases such as vitiligo, atopic dermatitis, and alopecia areata (23). In addition, circulating IL-18 has been linked to dyskeratosis and cell death in rashes associated with adult-onset Still’s disease (24). IL-18 is released from keratinocytes following UV light (25) and microbial stimuli (26), both of which have been linked to DM. Thus, IL-18 is an intriguing candidate for further study in the pathogenesis of DM skin lesions.

In summary, we have examined the gene expression changes in a large cohort of DM skin lesions. We have confirmed a high type I IFN presence, including IFN-κ, in DM skin. In addition, we have identified IL-18 as a uniquely elevated cytokine in DM lesions that in combination with *LCE2D*, *LCE1B*, *KRT80*, and *TPM4* expression cleanly distinguishes DM from CLE lesions, indicating this DM biomarker panel may be diagnostically useful, especially in patients with skin lesions who have not yet developed muscle disease. Prospective cohorts should test the utility of this diagnostic tool and whether it also has any prognostic value for treatment response or long-term outcomes for DM patients.

## Methods

### Sample acquisition.

For the inquiry cohort, 43 skin biopsies from 36 unique DM patients from 2013–2018 were identified via a diagnostic concept code search of the U-M Pathology Database using the search terms “dermatomyositis” and “skin.” Each case was reviewed in-house, and patients who met both clinical and histologic criteria for DM without overlapping evidence for systemic lupus were included in the study. Comparative cases of 43 SCLE and 47 DLE biopsies were identified as previously published (9, 27, 28). Data from CLE and DM microarrays are available through Gene Expression Omnibus GSE81071 (28) and Gene Expression Omnibus GSE142807, respectively. For the validation cohort, an additional 9 DM and 9 CLE (2 DLE, 1 acute CLE, and 6 SCLE) cases were identified from U-M 2018–2019 pathology records. Table 1 shows DM demographic information. Rash response to antimalarials was defined as notation of skin improvement by the provider and no escalation of therapy required for skin disease after antimalarial was started. Control skin biopsies were obtained through biopsy and from FFPE preservation as previously reported (9, 27, 28). Only 1 control overlapped between the DM and CLE data sets.

### RNA isolation and microarray procedures.

Five 10 μm sections were cut from FFPE blocks via a microtome. RNA was isolated using the E.Z. N.A. FFPE RNA Kit (Omega Bio-tek) according to the manufacturer’s instructions. Expression analysis was completed through the U-M Advanced Genomics core as previously described (28). Affymetrix Human Gene ST 2.1 array plates were used for transcriptional analysis. The microarray data were first processed by the Robust Multichip Average method (29), and quantile normalization was then applied. Mean average expressions were used for lesional skin samples from the same patient. There were 41 DM lesional skin (from 36 patients) and 5 control samples in the DM inquiry cohort.

### scRNA-Seq.

A total of 6 mm punch biopsies were taken from 1 female DM patient from both lesional and nonlesional skin (upper thigh), and nonlesional biopsies were taken from a female healthy control and incubated overnight in 0.4% Dispase (Life Technologies) in Hanks’ Balanced Saline Solution (Gibco) at 4°C. The epidermis was then digested in 0.25% Trypsin-EDTA (Gibco) with 10 U/mL DNase I (Thermo Fisher Scientific) with rocking for 1 hour at 37°C and quenched with FBS (Atlanta Biologicals). Dermis was minced and rocked in 0.2% Collagenase II (Life Technologies) and 0.2% Collagenase V (MilliporeSigma) in plain medium for 2 hours at 37°C. After digestion, the samples were strained through a 70 μm nylon cell strainer, washed with 5 mL RPMI, and counted. Epidermis and dermis cells were recombined at a 50:50 cell density (to prevent overwhelming the run with only epidermal cells), spun, and resuspended in 500 μL IMDM + 10% FBS for 10x Genomics processing. Single-cell 3′ libraries were generated using the 10x Genomics V2 protocols sequenced using the Illumina NovaSeq 6000 platform. For analysis, the Cell Ranger pipeline was used to conduct alignment and barcode and unique molecular identifier read counting. The uniform manifold approximation and projection technique approach was used for dimension reduction, and clustered cells were mapped to corresponding cell types by matching cell cluster gene signatures with putative cell type–specific markers.

### Real-time PCR.

A total of 100 ng RNA was reverse-transcribed into cDNA, followed by quantitative real-time PCR analysis by the DNA sequencing core at U-M on an ABI PRISM 7900HT (Applied Biosystems). Gene expression was calculated by fold change relative to lupus group. The human primers used were as follows (all listed 5′→3′): GAPDH GAGTCAACGGATTTGGTCGT (forward), TTGATTTTGGAGGGATCTCG (reverse); IFN-α (master primer) TCCATGAGATGATCCAGCAG (forward), ATTTCTGCTCTG ACAACCTCCC (reverse); IFN-β GCTTGGATTCCTACAAAGAAGCA (forward), ATAGATGGTCAATGCGGCGTA (reverse); IFN-κ GTGGCTTGAGATCCTTATGGGT (forward), CAGATTTTGCCAGGTGACTCTT (reverse); CD2 CTGAAGACCGATGATCAGGA (forward), CACAGGTCAGGGTTGTGTTG (reverse); CCL7 AAACCTCCAATTCTCATGTGGAA (forward), CAGAAGTGCTGCAGAGGCTTT (reverse); IL18 CCCTTTGCTCCCCTGGCGAG (forward), AGACTGCAGCAGGTGGCAGC (reverse); LCE1B AGGCTGCTGCTAAAGTGGAT (forward), TTTTGGGCCTCTGAACTCCA (reverse); LCE2D CCCAAGTGTACCCCAAAATGT (forward), TTCACTCTCACAGCAATCGGG (reverse); KRT80 CCTCCCTAATTGGCAAGGTG (forward), AGATGCCCGAGGTCGAAGAT (reverse); TPM4 GAGGTAGCTCGTAAGCTGGTC (forward), ACCGTTCTCTCTGCAAATTCAG (reverse); MX-1 TACCAGGACTACGAGATTG (forward), TGCCAGGAAGGTCTATTAG (reverse); and OASL CCATTGTGCCTGCCTACAGAG (forward), CTTCAGCTTAGTTGGCCGATG (reverse).

### IHC.

FFPE human skin tissues were sectioned and stained as follows: sections were deparaffinized; rehydrated; and heated at 100°C for 20 minutes in antigen retrieval buffer, pH 6, for treatment with IFN-α, IFN-β, and IL-18, whereas antigen retrieval buffer, pH 9, for IFN-κ and MX-1 was used. Slides were washed, treated with 3% hydrogen peroxide in PBS for 5 minutes, blocked, and incubated with anti–IFN-α, anti–IFN-β, anti–IFN-κ, anti-MX1, and anti-IL18 antibodies at 1:100 dilutions (Santa Cruz Biotechnology sc-80996, Abcam ab140211, Abnova H00056832, Abcam ab95926, and ORIGENE TA324190, respectively) overnight at 4°C. Appropriate negative (no primary or secondary antibodies or isotype control antibodies IgG [Abcam 125938], IgG2ak [Invitrogen, Thermo Fisher Scientific 14-4724-82], and IgG2bk [BioLegend 401201]) antibodies were stained in parallel with each set of the previously mentioned slides. All slides were then incubated with biotinylated secondary antibodies at 1:200 dilutions (Vector Laboratories goat anti–rabbit IgG biotinylated antibody PK-6101 and anti–mouse IgG biotinylated antibody PK-6102), followed by incubation with Vectastain ABC reagent, and stained with peroxidase substrate, counterstained with hematoxylin, dehydrated, and mounted. Images were acquired using a Zeiss microscope at indicated magnifications.

### Pathway, cell type, and literature-based network analyses.

Canonical pathways were identified using IPA software (QIAGEN). Significantly regulated genes were analyzed by creating biological literature-based networks using Genomatix Pathway System software (version v3. 110621) (https://www.genomatix.de). The function-word level was used as a minimum evidence level parameter. Analysis for enrichment of cell types was performed on the normalized data set of HC and DM genes using the xCell tool (http://xcell.ucsf.edu/) (11).

### Statistics.

Principal components analysis was conducted using inverse-normalized expression levels of all detectable transcripts for the microarray data. We then performed differential expression analysis using a linear model implemented in the limma package (30). The limma package utilized a modified 2-way *t* test with robust variance estimation computed using an empirical bases approach. False discovery rate ≤ 10% and |log_2_ fold change| ≥ 1 were used to declare significance. To identify candidate genes that serve as biomarkers differentiating the DM and CLE samples, we first used the microarray data sets as a training set, and then validated using independent samples. We applied 5-fold cross-validation to train random forest classifiers for genes that are only upregulated in the control versus DM comparison microarray but not in the control versus CLE comparison (restricted to genes with *P* > 0.5 and log_2_ fold change < |log_2_ 1.5|). Specifically, we applied the approach for 5 selected genes (from the previous list) involved in inflammatory response in the combined DM and CLE samples. We then validated the results by assessing the expression levels in independent DM and CLE samples.

### Study approval.

FFPE samples were acquired under IRBMED HUM00072843. Prospective patient biopsies for scRNA-Seq were obtained from participants of the U-M IRBMED HUM00066116. All patients gave written informed consent and met clinical diagnostic criteria for DM without overlapping evidence for systemic lupus.

## Author contributions

JMK, GAH, TN, JEG, and ACB conceptualized this study. LCT, MGK, ACB, GAH, TJR, LL, SNE, and GAH collected the data and/or specimens. LCT, MGK, CCB, MTP, RW, JEG, SNE, GAH, and JMK performed the data analysis. JMK, GAH, SNE, and MGK drafted the manuscript. All authors read, edited, and approved the final manuscript.

## Supplementary Material

Supplemental data

## Figures and Tables

**Table 1:**
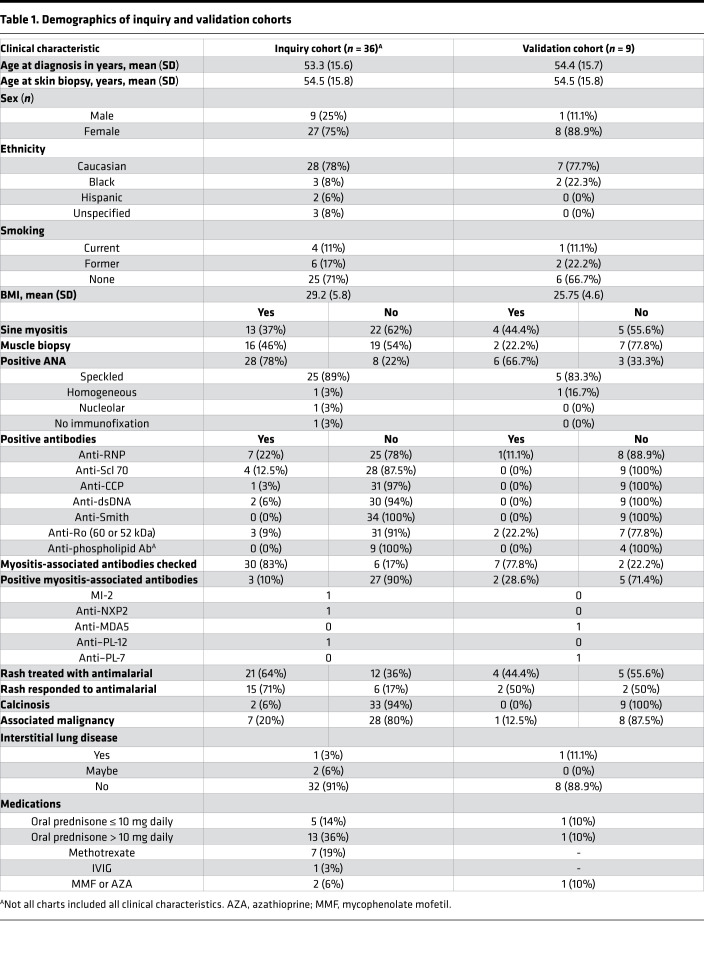
Demographics of inquiry and validation cohorts

## Version 1 07/09/2020

In-Press Preview

## Version 2 08/20/2020

Electronic publication
