# Supplementary material for: IL18-containing 5-gene signature distinguishes histologically identical dermatomyositis and lupus erythematosus skin lesions
Source: JCI Insight. 2020 Aug 20;5(16):e139558. doi: 10.1172/jci.insight.139558 (PMC7455118; doi:10.1172/jci.insight.139558)
Supplement: Supplemental data [file jciinsight-5-139558-s150.pdf]

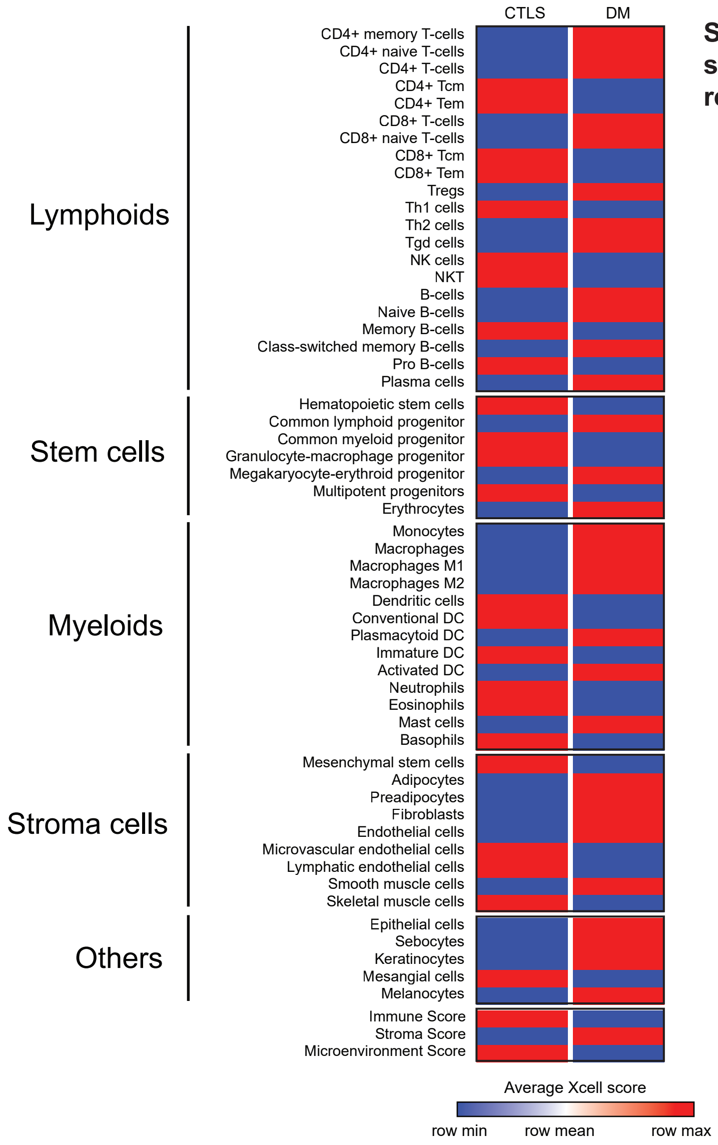

Supplemental Figure 1: xCell analysis of HC vs. DM showing stromal and inflammatory cell populations responsible for gene expression.

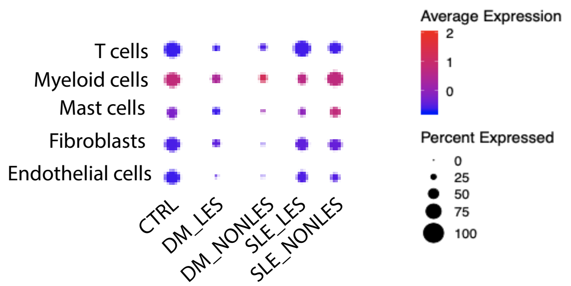

Supplemental Figure 2. Single cell RNA-sequencing analysis of IL-18 expression in inflammatory cell populations in control (CTRL), DM (lesional and non-lesional) and SCLE (lesional and non-lesional) skin. Graph represents gene expression in indicated cells for % of cells expressing IL-18 (by size of circle) and degree of expression (by color).
